# Beyond Individual Responsibilisation: How Social Relations are Mobilised in Communication About a Dementia Self-Testing App

**DOI:** 10.1007/s10728-024-00498-8

**Published:** 2024-12-27

**Authors:** Alexandra Kapeller

**Affiliations:** Department for Thematic Studies: Technology and Social Change, Linköping University, Hus T, Campus Valla, Linköping, 58183 Sweden

**Keywords:** mHealth, Self-testing, Dementia, Responsibility, Responsibilisation, Relationality

## Abstract

Research on mobile health (mHealth) applications has investigated how such technologies contribute to a responsibilisation of users/patients. This literature largely focuses on the individual responsibilities constructed by the apps and the neoliberal environments that enable the positioning of the user as responsible. With this focus, this scholarship is less attentive to the role of social relations in responsibilisation. In this article, I demonstrate how relational responsibilities are constructed in the communication of a North American self-testing app for “early changes in cognition”. Through an analysis of qualitative expert interviews and images on the app’s web presence, I show how social relations are, in fact, mobilised in the construction of the responsibilities to support the user in the test situation, to take the test for the sake of others, and to make others take the test. Based on this analysis, I argue that the role of social relations should receive more attention in the literature on responsibilisation, because they lead to additional, sometimes gendered responsibilities that a focus on individual responsibilities would miss.

## Introduction

Literature in critical digital health studies has problematised the role mobile health (mHealth) apps play in the shift of responsibilities from state to individual that has been called responsibilisation [1–3]. This shift is especially salient for commercial direct-to-consumer (DTC) self-testing apps which provide the indication of a diagnosis, formerly a responsibility and privilege of healthcare professionals [4, 5]. With these technologies, users are expected to take responsibility for testing, interpretating results and taking action [2].

In this important criticism, the analytical focus often lies on an individual user’s abilities and responsibilities but not on their social relations and which role they play in responsibilisation. This absence is surprising given the vast amount of literature in feminist bioethics and medical humanities demonstrating the importance to consider a patient’s social relations. This focus on individual responsibilities is also unfortunate as it makes literature on responsibilisation miss additional relational responsibilities that patients/users and their social relations are burdened with.

In this article, I analyse which role social relations play in the construction of responsibility. For doing so, I use the case of the mentioned North American self-testing app for “early changes in cognition”. The analysis includes qualitative interviews with experts involved in the app’s development and images from the app’s public website and Twitter page. I demonstrate how social relations are, in fact, mobilised in the construction of responsibility in the experts’ reasoning and in communication about the app. Relational responsibilities include supporting the user in the process of testing, taking the test for the sake of others, and being vigilant about cognitive changes in others and make them take the test.

Based on this analysis, I argue that recognising the importance of social relations in the construction of responsibilities is vital to the literature on responsibilisation. Doing so allows for a critique of the relational responsibilities that app users and their environment are burdened with in addition to the individual responsibilities already captured by the literature. In the bigger picture, the presence of social relations in the construction of the responsibility to test complicates the focus on the patient/user and their app currently employed by mHealth literature. In line with the suggestions by feminist bioethics scholars, it points to the necessity of employing a less individualistic conceptualisation of the self in self-testing and mHealth beyond.

## Background

Scholars in sociology of health have argued that mHealth technologies constitute a new form of the ‘patients as consumer’ idea [2]. What is new about mHealth apps is that the locus of healthcare potentially shifts from doctor’s office to living room: patients become users. This shift is worth examining as it is stated to involve a role change from the patient, a relatively passive healthcare recipient, towards the user, an active participant in the care process [6]. This user is conceptualised as a particular subject: health-literate, empowered, responsible. Underlying this conceptualisation are criticised to be neoliberal ideas about an “ideal-type responsible citizen” [3] who “makes rational choices and is independent, self-reliant and responsible” [7]; a “responsibilised self” that is supposed to control and manage their body [8]. As Lupton summarises:

Discourses of the digitised and digitally engaged patient may be viewed as part of a neoliberal political orientation to patient care and preventive health. They position lay people as ready and willing to actively engage in their own healthcare and promote their own health, in the attempt to *shift the burden of such responsibilities from the state to the individual*. [2, emphasis added]

For Lupton and others, engaging patients/users in (preventive) health practices is not an innocent move. It shifts responsibilities from healthcare systems to the user: health becomes a private issue instead of a collective one. The shift from “My health is the responsibility of my physician” to “My health is my responsibility, and I have the tools to manage it” [9] has been captured by the term ‘responsibilisation’, a term with origins in literature on governmentality [10]. Criticism of this shift of responsibility includes the concern that framing health as an individual problem may distract from environmental factors on health that cannot be individually resolved [11]. Placing responsibility for health on individuals is criticised as unrealistic given that health is not completely controllable, that many people cannot or do not manage their own health [1, 12–14], and that many rely on informal caregivers [15]. Hence, responsibilisation entails presenting citizens with unrealistic, overwhelming and burdensome tasks – tasks whose unsuccessful completion may result in victim-blaming [7, 16], going as far as blaming people for falling ill:

Pushing the argument to its extreme, this may also mean that through the life choices made, individuals become personally, albeit still partially, responsible for their trajectory towards the development of AD [17].

The responsibility not to fall ill is usually constructed as an individual one, especially in communication about preventive health and self-testing. Not falling ill is also part of the relatively new rhetoric and responsibility to “age successfully” [18, 19], for which technologies provide new tools [20]. In line with the neoliberal conceptualisation of the user mentioned above, following up on these responsibilities is something people do by and for themselves – independently, autonomously, empowered [13, 21]. A study into information provided by dementia organisations has demonstrated how the information on their websites positions readers as at-risk and individually responsible [22] through what has been called the doctrine of “methodological individualism” [23]. This doctrine centres the individual and explains social phenomena of health with individual agency, e.g., by highlighting the importance of lifestyle changes [23]. In this responsibilisation, technologies like mHealth can play a crucial role [2, 24]. With the individual responsibility to take care of one’s own health, and the availability of self-tests, the personal choice to test is turned into a social obligation [17]. Self-testing apps exemplify responsibilisation perfectly, as they put responsibilities traditionally reserved for healthcare professionals into the hands of a user, namely the responsibilities to decide when to test and to interpret the result correctly [4, 5].

In the context of *dementia* self-testing, responsibilisation is further fuelled by the medical discourse on early dementia testing. Early detection of dementia is named as a priority in most (inter)national dementia policies [25], for three main reasons; (1) it would rule out treatable conditions [26], (2) it could help patients to prepare for the future and to receive optimal care [27] and (3) it could enable patients to make lifestyle changes so that the course of disease is slowed down [e.g. 28]. This medical imperative to test serves to legitimize the importance of early detection as portrayed in media [27, 29], online information about dementia (Lawless et al., 2018) or online AD tests [17]. Interestingly, This the discourse around this imperative is usually flattened when it is used to emphasize the importance of early detection. Although there seems to be consensus on the importance of testing in medical literature, there is less consensus on the preferable way and time to test [30]. For example, it has been argued that instead of an *early* diagnosis, e.g. through screening programs, health authorities should strive to promote *timely* diagnoses, i.e., at a time that considers the patient’s preferences and situation [31, 32]. This nuance is usually absent when the importance of early detection is highlighted.

Although literature on responsibilisation criticises the individualistic conceptualisation of subjectivity that underlies responsibilising interventions in mHealth, surprisingly, it rarely attends to relational responsibilities that they co-create. As an exception, Kearns et al. [33] have argued that the information provided by self-testing apps could “have serious implications for others and raises questions about the responsibilities of the person towards others” [33]. In the context of depression guidelines, McPherson & Oute have criticised the responsibilisation agenda behind carer involvement recommendations [34]. Responsibility towards others has also been examined in other contexts, e.g. genetic diseases, where the disclosure of test results is discussed as a possible responsibility towards others [35] or more abstractly in connection to relational ethics of apps [36]. Such relational responsibilities could be expected to be studied given the large body of literature in medical humanities and feminist bioethics that, in examining autonomy, has argued against an individualistic conceptualization of the self [37, 38], and that has demonstrated how patients decide, suffer and organise themselves together with others [35, 39–41]. Yet, these relational responsibilities have not received much attention in the literature on responsibilisation.

## Method

As mentioned in the introduction, the case study for this article is a North American app that tests for early changes in cognition. The indication it provides is hoped to ultimately contribute to an early detection of mild neurocognitive disorder or dementia. I chose this app because it is scientifically validated (so concerns about its scientific quality can be precluded), because it has been on the market for a few years and downloaded in substantial numbers (so there would be some user experiences to ask about), and because it exemplifies the trend toward early detection in (mobile) healthcare.

For this article, I used two kinds of empirical material: I held four qualitative in-depth expert interviews with researchers and developers affiliated with the app company, as well as analysed images from the company website and their Twitter account, i.e., public websites. In combination, the analysis of this material helps me explore how social relations are brought up when experts talk about the app and how they are mobilised in the presentation of the app online.

### Interviews

This article is part of a larger study of this app involving interviews not just with experts (researchers and developers) but also with users. This study was conducted in accordance with the Declaration of Helsinki [42]. Of ten experts, whom I contacted by email or phone, six responded positively and four declined or did not reply. With these six people, I conducted six initial interviews, which informed whether the study was worthwhile but were not used in the analysis.

After these initial interviews, I applied for the necessary ethics approvals for the actual expert interviews and the user interviews. [Country redacted for blind peer review] research ethics approval was sought and received for both (Ref. no. 2020–04970). Research ethics approval for expert and user interviews was also sought from the ethics board at a US-American university since the user part of the larger study was to be conducted from the United States. The ethics board stated that the research did not require review and that they agreed to the author conducting user and expert interviews (Office of Responsible Research Practices; decision 2021-01-29).

After receiving the ethics approvals, I held four expert interviews in May-June 2021, via Zoom. Questions tended to the characteristics of the app users, to whom the app would and would not work for, to reasons for developing and using the app, and to how marketing strategies are informed. Interviewees received an information sheet and gave written consent to take part in the interview. All interviews were semi-structured, lasted between 30 and 120 min, and were audio-recorded and transcribed. All names and other identifying information were redacted or replaced by pseudonyms.

For my initial analysis, which led to this article, I followed a thematic analysis. I chose this method because thematic analysis is theoretically flexible [43] and because it is concerned with meaning and meaning-making as “context-bound, positioned and situated” [44], which helped me reflect on what I was interested in, namely how the interviewees talk about their app and early detection, and which meaning they assign to the practice of test-taking. In this initial phase, I semantically coded the transcripts, constructing themes and subthemes [44], with the software f4analyse. Themes included, among others, the description of a responsible user, their goals with the app, and the importance of early/timely detection. Although I did not initially identify it as a theme or subtheme, I noticed that many quotes in particularly these themes engage with social relations. This focus stood out to me as a contrast to the responsibilisation literature.

To explore the role of social relations in responsibilisation for this article, I went back and forth between theory and further analysing the material. I searched for text passages in the interview material that mentioned keywords such as “family”, “spouse”, “partner”, etc. By that time, I knew my interview material well and was sure that I included all quotes referring to social relations. These quotes were examined with attention to how the interviewees positioned the test, dementia, and users and their relatives. When examining the quotes, images from the app’s website and Twitter page, which I had visited in the begin of the project, came to my mind. I was interested in how these images would relate to the subthemes of the expert interviews. Given the importance of studying how visual content helps to frame health-related messages [45], I decided to include this material in the analysis.

### Images

My image analysis started with the assertion from visual discourse analysis that visual material is “ever disciplining and never innocent”, as it tells us “who we ‘should’ be and what we ‘should’ do” [46]. I was interested in how the images, including their depiction of social relations, contribute to the construction of a responsible self (who we should be) and responsible actions (what we should do) in connection to dementia testing. I followed visual discourse analysis in that I examined content, referents, and context of the images [46]. First, I described what the pictures depict and then examined the colours, the portrayed relationship between the depicted people, my impression of the mood the image evokes, and where the image is published to conclude if and how the images contribute to the construction of responsibilities.

The material was chosen not with the idea of providing an exhaustive list of items that refer to social relations, but to portray an illustrative collection of how social relations are engaged with on the app company’s public communication outlets. The website, excluding its blog section, contains roughly ten images that depict people. Of these ten, three images show at least two people together, while the others depict a single person taking the test or just a hand holding a tablet. Of these three, I chose two because they take up prominent space on the website, one being the banner of the homepage. Two more images were chosen from the company’s twitter page, which shows hundreds of tweets with various images. I chose them because they either mention other people than the prospective user or their images depicted more than one person; and because they do so in a particularly clear way. As this material is publicly available and does not contain any personal data, ethics review for the image analysis was not necessary. Using the images in this text is in accordance with copyright law, as using copyrighted material for scientific presentation is permitted, equivalent to the “fair use” and “fair dealing” in North America. For confidentiality reasons, I did not include a link to the websites the images were taken from and a reverse image search confirmed that the origin of the pictures is not definitely to determine.

Together, the interviews and the online material combine social relations and responsibility in three instances: “responsibilities in the test environment”, “responsibility to others to take the test”, and “responsibility to make others take the test”. In the analysis, I examine these findings against the backdrop of the literature on responsibilisation and mHealth presented in the Background section.

## Analysis

The analysis will be presented parallel to the identified subthemes. I will first point out how the depiction of the test environment on the app’s website visually introduces social relations. Second, I demonstrate how interviewees and images construct responsibility to test oneself towards others. Third, I present how the responsibility to be vigilant about other’s cognitive health and make others take the test is established.

### Responsibilities in the Test Environment

**Fig. 1:**
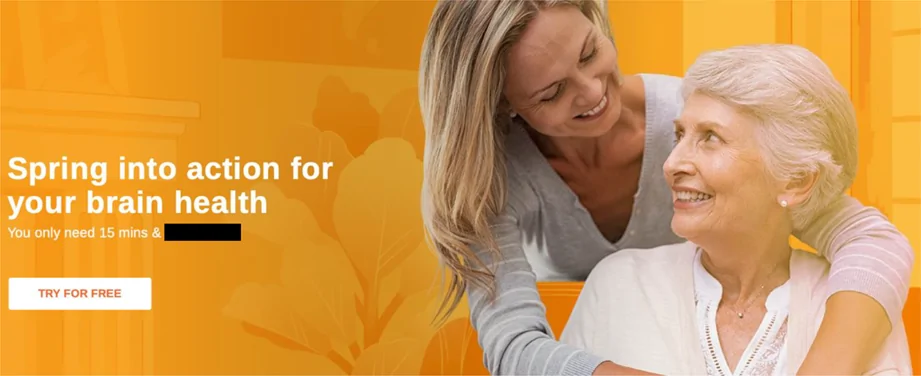
Screenshot of the app’s homepage, taken on 5 November 2021

When opening the app’s website, the reader is first presented with a large image and a few lines of text (Fig. 1).

The text is addressing the reader with an imperative, in bold letters: “Spring into action for your brain health”. Research on similar formulations (“Keep your brain active!”) in the communication of dementia prevention has pointed to an effect of such imperative formulations: they “establish the necessity of participation in dementia risk-prevention practices, and position audience members as needing to take personal responsibility for dementia risk management” [22]. The imperative, which is the eyecatcher on the website, is followed by advice on how to follow through: “You only need 15 min and our app” – you can even “try for free”. At first sight, the image’s text, which tells the reader what to do, seems to fit seamlessly into the literature discussed in Background. The medically informed imperative to detect early gives the app its purpose and urgency. This urgency is portrayed unquestionable: critiques of early detection, such as the discussion of early or timely detection or the problem of false positive results, are absent. Further, the methodological individualism in mHealth, in which the responsible subject makes autonomous decisions and takes care of their brain health [3, e.g. 23], explains why the user is the recipient of this imperative. The reader is addressed as a consumer, who makes a choice on which health product to purchase. The result is an individual responsibility for managing one’s brain health, and the test is positioned as the tool to do so [9].

Besides this unsurprising expression of individual responsibility, the picture also introduces something new, namely a second person next to the one taking the test. Figure 1 shows a sitting elderly person. A middle-aged person stands behind them and gently puts their arms around them from behind. The two are exchanging smiles and I immediately think of them as adult child and parent. It evokes the idea of care, and the mood seems light and loving. This togetherness, i.e., just the fact that *two people* are depicted, complicates the idea of individualised responsibility – and who is meant by “your/you” in the text. Who springs into action for whom? Should the elderly person take the test for their own and her child’s sake? Should the child make the parent take the test? Should they take the test together? Fig. 1 evokes questions about relationality.

**Fig. 2:**
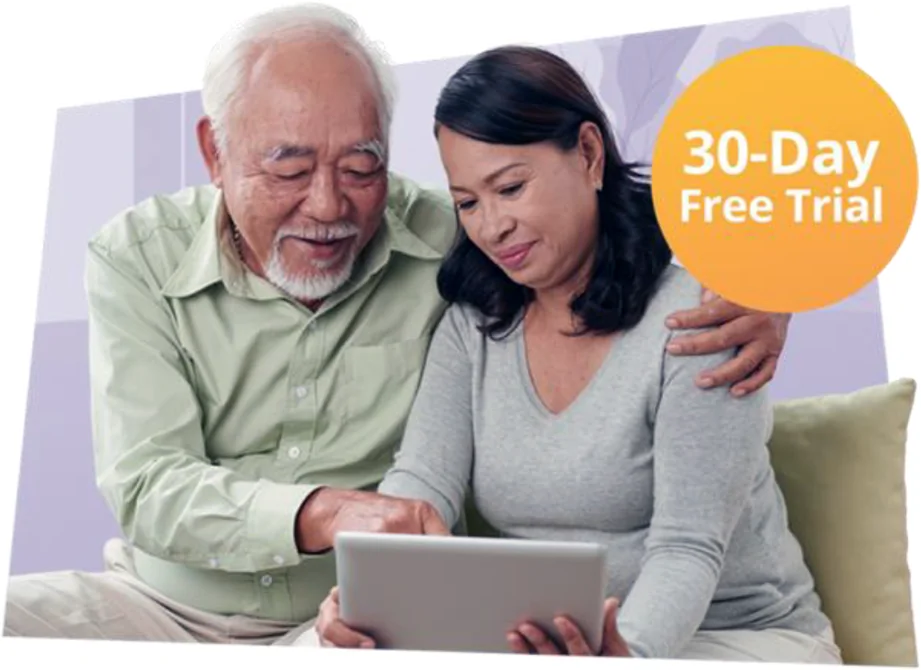
Picture from the website, taken on 5 November 2021

Also Fig. 2 below, taken from further down on the website, shows two people. A younger person is holding and looking at a tablet, while an elderly person sitting to their right is putting their left arm around the younger person’s shoulder and using their right hand to operate the tablet. Presumably, the younger person showed the app to the elderly one, and the elderly person is filling out the test.

Here, the two people are depicted together even while one is taking the test. This constellation is surprising because there are reasons to take the test alone. First, doctors or family members who know about the test might be inclined to “help” the user fill out the test, leading to incorrect results. Accordingly, taking the test in private avoids administrator bias or “cheating”, which is a selling point of a self-administered test. Here, relatives would have the responsibility to refrain from intervening in the test.

Besides accuracy, the second selling point of self-testing apps is privacy, i.e. the ability to take it alone and control over whom to tell about the test and the result. Accordingly, the instructions in the app recommend the test be taken in a “comfortable and quiet environment”. The experts are well aware that dementia is a stigmatised disease, and that many people refrain from discussing it with their doctors and loved ones:

Well, people fear the disease. They are afraid of talking about it. It is hidden within the family. They don’t go to the doctor and if they do, the family doctor, the GP, doesn’t always respond very well. So, they have trouble getting a diagnosis. (Peter)

[With the app, ] You don’t have the stigma, don’t have to go in and admit to somebody that you’re forgetting things and you’re feeling concerned. (Elliott)

These quotes background the individual responsibility to test and foreground relationality as the reason for taking the test via an app: users take the test by themselves because they are afraid of being stigmatised by others.

Yet, Fig. 2 suggests the idea that taking the test can be a shared activity and that a loved one in the room may contribute to the comfort mentioned in the instructions. In this sense, the aforementioned question of who springs into action is answered: it is the relative who springs into action for the elderly person, exercising their responsibility to comfort them. The success of a test is then not an individual responsibility but also dependent on the correct behaviour of the additional person present: to comfort but not to intervene.

In sum, website and interviewees construct the test situation itself not as a moment of actualised independence and control, but as a moment in which the user reacts to and depends on social relations.

### Responsibility to Others to Take the Test

This section introduces how the responsibility to get tested is constructed as an individual responsibility *to others*. In the interviews, experts often mentioned other people and social relations as reasons to why one should get tested. Besides personal well-being, the hardships associated with having and caring for family members with dementia are reasons to postpone the diseases’ later stages as far as possible – much in agreement with the medical literature on early detection [e.g. 47]. Experts emphasised that recognising the disease early may help family members prepare, an opinion that is also communicated via the website: In a post on reasons why people should test for Alzheimer’s disease, the author lists improved treatment options, the ability to make choices for oneself, and the mental health of one’s family. Taking a test early would, in the case of a positive result, give family members time to learn about Alzheimer’s and support mechanisms, and in the case of a negative result, provide relief by “easing their concerns”.

Mentioning family and loved ones appeals to a person’s wish to not harm those around them: even if you don’t care about yourself, care about others! Note also how this post mentions Alzheimer’s, not “early changes to cognition”. The medically and socio-culturally undesirable later stage of dementia can be interpreted as serving as an incentive to test early, but with a new twist: the imperative to test for your own sake is extended to the imperative to test for the sake of others. Because the disease will make it difficult to maintain relationships and because others will have to “carry the care burden”, social relations are positioned as at risk with dementia:

And the way people feel treated, they lose family and friends, and contacts, if they get dementia. Caregiver burden is the same everywhere, maybe even worse in developing countries. (Peter)

The implied message is that, by following up on their responsibility to detect early, people can maintain their social relations longer and reduce the burden on others: Take the test if you want to keep your relationships. This implicit positioning of the test as a safeguard for relationships resembles results from previous research on an Alzheimer drug advertisement, which depicts an elderly couple [48]. According to the analysis, that advertisement suggests a responsibility to each other for taking the Alzheimer drug so as to not be a burden to each other. The drug is positioned as enabling the couple to live up to this responsibility. Along the same lines, the self-testing app is discursively positioned as the key element in maintaining relationships instead of losing them to dementia.

### Responsibility to Make Others Take the Test

**Fig. 3:**
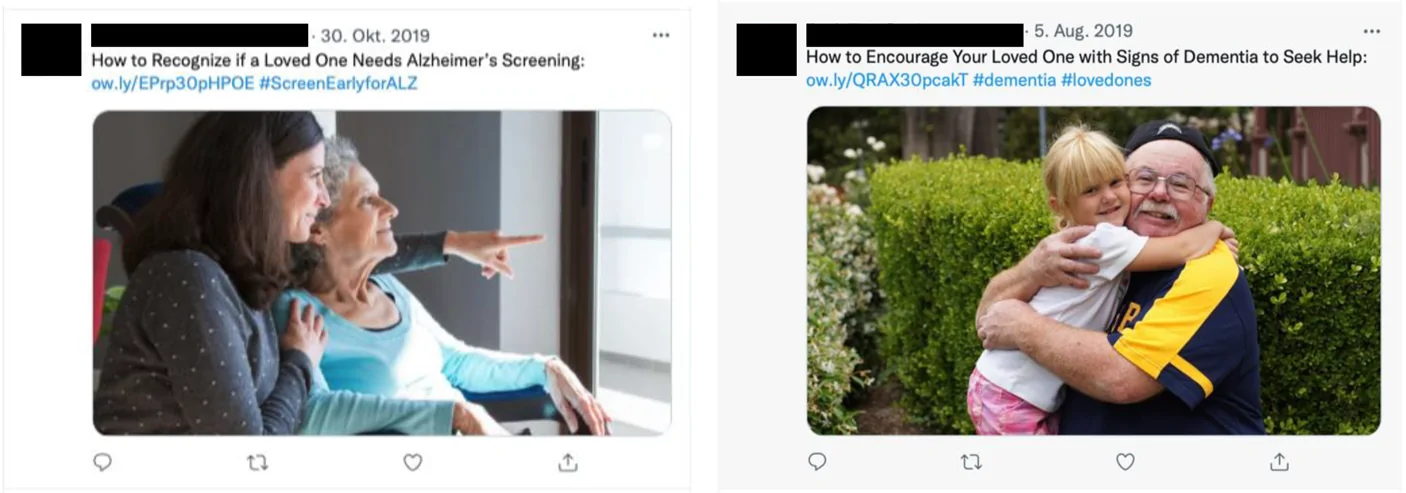
Tweets from 2019, screenshots taken on 5 November 2021

In the material, responsibility towards others does not only mean the user’s own responsibility towards others. Consider the tweet on the left in Fig. 3 below, which contains, again, a picture of a younger person sitting behind an elderly person. Both are smiling and looking at a window, and the younger one has one arm around the elderly person, using their hand to point towards the window. By pointing, the younger person seems to be giving directions to the older person, or explaining something to them. Because of their proximity and body contact, the two people appear to have a close relationship. The image is complemented with text above it: “How to Recognize if a Loved One Needs Alzheimer’s Screening”.

The text, mentioning a “loved one”, confirms the close relationship between the two people. As the title suggests, perhaps the younger person recognised that their parent needs Alzheimer’s screening and is pointing them in this direction. The second tweet in Fig. 3 shows a grandparent and their grandchild, hugging each other and smiling. Above the image, the text says, “How to Encourage Your Loved One with Signs of Dementia to Seek Help”. The two headings indicate that the tweets do not appeal to a person who is concerned about their own, but about others’ cognitive health, i.e., all people who may be concerned about someone else’s cognitive health. Addressing relatives directly serves a purpose. The aforementioned research on Alzheimer drug advertisement explains how addressing relatives constructs the disease and the drug as a concern for more people, which widens the range of potential customers [48]. Accordingly, the app also reaches a larger target audience as it becomes an interesting product for more people. This expansion evokes a shift in responsibility from the individual user to their social environment. Literature suggests that relatives are increasingly responsible for detecting signs of cognitive impairment; they “spring into action” for their family members. As Egher and Wyatt state:

The ascription of health-related responsibility becomes a bottom-up approach nuanced by the state of one’s cognitive abilities: as soon as suspicion arises that they may have severely deteriorated, the next-of-kin are called upon to engage in diagnostic practices. The availability of such tests is built upon certain normative expectations regarding family and friendship, as relatives or friends are assumed to respect the autonomy of the person and act in his/her best interest [17].

Hence, the app not only includes relatives as a target audience for advertisement, it also ascribes new responsibilities to them. The “normative expectations” in the quote refer to the relatives’ double responsibility to both recognise if their family members show signs of cognitive changes and to take action in an ethical way. One expert explains that while relatives are often the first ones to notice cognitive changes, they do not always act:

The family members who know it, they aren’t calling up the patient’s doctor and saying you know ‘My wife or my husband is losing it’, you know. It takes more effort. (Alan)

I was very frustrated at the patients that would come into my office that had symptoms for three, four, five years. […] I’m going ‘If only they had been coming two or three years ago!’ You know? Soon after the cognitive changes were noticed by others. You know, these were the time when they were actually noticed by other family members or themselves or someone. (Alan)

In these quotes, the consequences of relatives failing to follow their responsibility to speak up relate to the medical reality of the “late stage”, in which treatment options are said to disappear. In turn, if relatives recognise cognitive changes and a diagnosis is obtained more quickly, there are better treatment options. Overall, the tweets and interview quotes in this section go beyond individual responsibility by constructing the responsibility to recognise signs and make others take the test. This responsibility was presented as a gendered one by the interviewees, for example:

We do know that women are much more likely to be interested in this, they tend to be the, I think, the… the… the pers- the individual in a family setting that takes the most ownership over health of members of the family. That’s why when you look at pharmacy, they’re very hell-bent on attracting women as new customers because they buy most of the pharmaceuticals and stuff for the house that pharmacies sell. […] they also are the ones that get their husbands to the doctors when they think there’s something going on, their kids, etc. So they tend to be the leaders of health care within the family unit. (Robert)

In a (heterosexual) family unit, the expert states, it is mostly women, or, as another participant called them, the “Chief Medical Officers” (CMOs), who make decisions about the healthcare of their family members. The term “CMO” was coined by a pharma-sponsored report, which explains that women “comprise the industry’s core consumer segment, as they set the health and wellness agenda for themselves and others, choose treatment regimens, and hire and fire doctors, pharmacists, and insurance providers” [49]. That women take on more health responsibilities is not a new observation. Feminist scholars have been pointing out an imbalance in health responsibility for a long time [50, 51]. This imbalance also concerns dementia, considering two thirds of informal AD caregivers in the United States are women [52]. It is then mostly women who seem responsible for recognising signs for dementia in others, for making them take a test, and for taking care of their relatives with AD.

## Conclusion

In this article, I used the example of self-testing apps, an emerging phenomenon in mHealth, to understand the construction of relational responsibilities. The analysis demonstrated how both interviewees and online material employ social relations when health responsibilities are constructed: to act correctly in the test situation, to take the test, and to make others take the test. “Spring into action” – also for others, not just for yourself. The urgency to do so is fuelled by the unquestioned medical imperative to detect dementia early.

These results are helpful for the discussion on responsibilisation insofar as they expand the “catalogue” of responsibilities that patients/users and their social environment are burdened with. The case study shows how these “new”, sometimes gendered responsibilities remain unattended to when studying only individual responsibilisation. Hence, it invites scholars working on responsibilisation to take seriously their own relational conceptualisation of the self when analysing constructed responsibilities in mHealth and beyond. Identifying how exactly health responsibilities are constructed within and through social relations could help criticising the gendered demands put on users and patients in the name of empowerment and hopefully aid in lowering these expectations. This project would be greatly advanced by future user studies on how users make sense of the responsibilities presented to them in advertisements and while using the app.

In public health, attention to the relational responsibilities could also add to the discussion on early or timely detection. Considering a user’s social relations can be important when determining the right point in time for testing – for example, if a relative is concerned or if the user is taking care of someone else. When determining this point in time, tit is worth considering that taking the test may have significant consequences for the user *and* their social environment.

Finally, the findings show an inconsistency in the technical and medical mHealth literature. While upholding an individualistic, neoliberal idea of the self, new responsibilities explicitly employ a user’s social relations. Scholars working on mHealth products would benefit from a relational conceptualisation of the self, as it would allow them to understand a user’s socially embedded situation, and hopefully to adjust their claims of individual control and empowerment over health [53, 54]. Adjusting these claims could enable a more fruitful conversation about the benefits of mHealth technologies.
